# Near-Infrared Spectroscopy as a Process Analytical Technology Tool for Monitoring the Steaming Process of *Gastrodiae rhizoma* with Multiparameters and Chemometrics

**DOI:** 10.1155/2020/8847277

**Published:** 2020-11-04

**Authors:** Yamin Zuo, Jing Yang, Chen Li, Xuehua Deng, Shengsheng Zhang, Qing Wu

**Affiliations:** ^1^School of Basic Medical Sciences, Hubei Key Laboratory of Wudang Local Chinese Medicine Research, Hubei University of Medicine, 30 Renmin South Rd, Shiyan, Hubei 442000, China; ^2^School of Basic Medical Sciences, Wuhan University, 299 Bayi Rd, Wuhan, Hubei 430072, China; ^3^Innovation Laboratory, The Third Experiment Middle School, Guizhou Key Laboratory for Information System of Mountainous Areas and Protection of Ecological Environment, Guizhou Normal University, 116 Baoshan North Rd, Guiyang, Guizhou 550001, China

## Abstract

Steaming is a vital unit operation in traditional Chinese medicine (TCM), which greatly affects the active ingredients and the pharmacological efficacy of the products. Near-infrared (NIR) spectroscopy has already been widely used as a strong process analytical technology (PAT) tool. In this study, the potential usage of NIR spectroscopy to monitor the steaming process of *Gastrodiae rhizoma* was explored. About 10 lab scale batches were employed to construct quantitative models to determine four chemical ingredients and moisture change during the steaming process. Gastrodin, *p*-hydroxybenzyl alcohol, parishin B, and parishin A were modeled by different multivariate calibration models (SMLR and PLS), while the content of the moisture was modeled by principal component regression (PCR). In the optimized models, the root mean square errors of prediction (RMSEP) for gastrodin, *p*-hydroxybenzyl alcohol, parishin B, parishin A, and moisture were 0.0181, 0.0143, 0.0132, 0.0244, and 2.15, respectively, and correlation coefficients (*R*_*p*_^2^) were 0.9591, 0.9307, 0.9309, 0.9277, and 0.9201, respectively. Three other batches' results revealed that the accuracy of the model was acceptable and that was specific for next drying step. In addition, the results demonstrated the method was reliable in process performance and robustness. This method holds a great promise to replace current subjective color judgment and time-consuming HPLC or UV/Vis methods and is suitable for rapid online monitoring and quality control in the TCM industrial steaming process.

## 1. Introduction

*Gastrodiae rhizoma* (*G. rhizoma*), also called “Tianma” in Chinese, which is regarded as one of the ten “magical plants” in China, has been widely used to treat diverse disease including headache, migraine, dizziness, epilepsy, infantile convulsion, tetany, and so on [1]. Phytochemical studies of *G. rhizoma* validated that the major chemical constituents linked with the pharmacological activity of this plant are phenolic compounds, such as gastrodin, gastrodigenin, bis (4-hydroxybenzyl) ether, 4-hydroxybenzaldehyde, and parishin, and more than about 30 phenolic compounds have been successfully isolated or transferred from *G. rhizoma* [2–5]. According to the Japanese and Chinese Pharmacopeia, the steamed roots of *G. rhizoma* have been defined as the standard form [6, 7], which is also defined as monarch drug or the major effective ingredient of many Chinese patent medicines, such as “Quantianma Capsules,” “Tianmaduzhong Capsules,” and “Tianmasu Tablets.” Therefore, the steaming process is a vital unit operation that affects the quality of the pharmaceutical products.

The purpose of steaming is to change the property of medicine and expand the range of medicine usage, to reduce side effect, or to be convenient for sliding pieces [8]. The endpoint of the traditional steaming process usually pays attention to only the content of gastrodin in *G. rhizoma* but ignore other active ingredients during the evaluation of quality, which may not be able to reflect the changes of the content of active ingredients and judge the endpoint accurately. Various instrumental techniques and methods have been developed for the qualitative and quantitative analysis of *G. rhizoma* constituents, including high-performance liquid chromatography (HPLC) or LC-MS, gas chromatography-mass spectrometry (GC-MS), and capillary electrophoresis (CE) [9–12]. However, these methods are often time-consuming, laborious, and tedious, since they require multiple steps of sample preparation. Therefore, new approaches that can overcome these drawbacks are highly desirable. Process analytical technology (PAT) tool which can increase the efficiency of process environment and guarantee the final product quality to be homogeneous and controllable should be paid attention, which is useful to monitor the content of the main active components changes in the steaming process of *G. rhizoma*.

Near-infrared (NIR) spectroscopy fulfills many of the criteria of an ideal PAT tool for pharmaceutical applications and has already been validated for different applications such as blend homogeneity, extracting, or active content and moisture determination [13–17]. As NIR spectra are featured by broad and overlapping absorption bands, which have thousands of wavelength variables, identification to specific chemical group vibrations seems to be rather difficult. Consequently, chemometric tools such as mathematical pretreatments and some regression methods are used to extract the significant and useful information. To the best of our knowledge, there have no reports yet on NIR spectroscopy as a PAT tool to monitor the steaming process of *G. rhizoma* so far.

For the qualitative and quantitative analyses by NIR spectroscopy, multivariate calibration models could be established through the combination of information-rich spectroscopy and efficient regression tools provided by modern mathematics. However, the selection of wavelength/variable is of great significance to acquire robust models with good performance. Nowadays, there are many mathematical strategies for variable selection such as stepwise multiple linear regression (SMLR), partial least squares (PLS), synergy interval PLS (Si-PLS), and principal component regression (PCR) [18–20], and some studies have shown that models built with effective wavelengths have a better performance.

The aim of this study was to (1) prepare different algorithm and build high-performance NIR calibration models in the steaming process of *G. rhizoma*: the PLS models will be evaluated to determine the contents of gastrodin, *p*-hydroxybenzyl alcohol, parishin A, and parishin B; the PCR models will be tested to determine the contents of the moisture content. (2) Investigate the feasibility and application of NIR spectroscopy to monitor the changes of chemical and physical properties mentioned before during the additional steaming process of *G. rhizoma*.

## 2. Materials and Methods

### 2.1. Materials and Reagents

Totally, 13 batches of raw *G. rhizoma* samples were provided by Guizhou Jiulong Group (Guizhou, China). Reference standards of gastrodin (batch No. 110807–201608), *p*-hydroxybenzyl alcohol (batch No. 111970–201702), parishin A (batch No. 62499-28-9), and parishin B (batch No. 174972-79-3) were purchased from China Food and Drug Testing Institute (Beijing, China). Methanol and acetonitrile were purchased from Kemmo Chemical Reagent (Tianjin, China). Water was purified by a Milli-Q water purification device (Millipore, USA). Other chemical reagents were all analytical grade.

### 2.2. Steaming Process and Sample Collection

Steaming process of *G. rhizoma* was simulated according to the actual process condition. Upon arrival, each raw *G. rhizoma* was washed cleanly immediately and then smashed into fluid homogenate. About 140 g of *G. rhizoma* was put into an electric steamer with a stirring paddle (Supor Group, Hangzhou, China) and heated for 10 minutes. The temperature of the herbal medicine during the steaming process was controlled at 100°C. The uniform distribution of samples with high or low concentrations is indispensable with the purpose to obtain similar prediction accuracy [21]. Samples were collected at 30 s intervals for reference analysis immediately after spectral measurements during the whole steaming process. In this study, about 200 samples were collected from 10 different batches, which were used to build the calibration and validation models.

All the procedures were strictly controlled to lower the risk of the uncertain parameters during sample collection, separation, and process.

### 2.3. Near-Infrared Spectra Acquisition

The NIR spectra were collected using an Antaris II FT-NIR analyzer (Thermo Nicolet, USA) at room temperature using the standard method. An integrating sphere diffused reflection mode with an InGaAs detector was selected to record the in-line NIR data throughout the steaming process dynamically.

Spectra were dynamically recorded in-line over the wavelength range from 4,000 to 12,000 cm^−1^ with a resolution of 8 cm^−1^. Each spectrum was an average of 32 scans and recorded as the logarithm of the reciprocal reflectance, which is log (1/*R*). The background spectrum was scanned with air as the reference in order to eliminate the effect caused by background. Considering that room temperature and relative humidity may be a risk to influence the surface moisture of *G. rhizoma*. Room temperature was maintained at 25°C and humidity at 80% during the spectra collection.

### 2.4. Sample Preparation and Reference Assays for HPLC

HPLC reference analysis was performed immediately after the sample NIR spectra were collected. The sample preparation was similar to the method used before [22]. About 0.5 g *G. rhizoma* sample was weighed and extracted with 25 mL of diluted alcohol, weighed the total solution content before ultrasounded for 30 minutes. Then, weighed again and complemented loss weight and filtered the solution twice, pipetted 10 mL of the subsequent filtrate to the evaporating dish that concentrated solution to dry, using the mixed solution of acetonitrile : water (3 : 97) to resolve residue, and fixed the volume to a 10 mL volumetric flask. The extraction solution was filtered with a 0.45 *μ*m filter membrane before HPLC analysis. We developed an HPLC method for the quantitative determination of gastrodin, *p*-hydroxybenzyl alcohol, parishin A, and parishin B in the samples. The chromatographic analysis was carried on a Diamonsil C_18_ column (250 mm × 4.6 mm, 5 *μ*m) at 30°C on a 1260 HPLC system (Agilent, Santa Clara, USA) consisting of a vacuum degasser, a thermostatic column compartment, a quaternary pump, an auto sampler, and a diode array detector (DAD). The mobile phase in this study consisted of 0.05% phosphoric acid solution (A) and acetonitrile (B) at a flow rate of 1.0 mL/minutes. A gradient program was set as the following profile: 0–10 minutes, 3%–8% B; 10–18 minutes, 8%–12% B; 18–40 minutes, 12%–25% B; and 40–50 minutes, 25%–40% B. The detection wavelength was set at 270 nm, and the injection volume was 10 *μ*L. The moisture content during the steaming process was determined by the weight loss method according to the Chinese Pharmacopoeia. All of the results acquired above were used as reference values for the NIR analysis.

The standard stock solutions used were prepared in advance by dissolving the four reference standards in 50% methanol (methanol: water = 1 : 1, v/v) to a final concentration of 0.0591 mg/mL for gastrodin, 0.0055 mg/mL for *p*-hydroxybenzyl alcohol, 0.0315 mg/mL for parishin A, and 0.0025 mg/mL for parishin B.

### 2.5. Spectra Transformation and Data Analysis

It is necessary to transform the NIR spectra to remove noise and irrelevant information and to select the variables to reduce the phenomena of redundancies and colinear besides to improve the prediction performance of the models. Therefore, spectra were transformed with several different methods, such as multiplicative scatter correction (MSC), standard normal variate transformation (SNV), first derivation (FD), and Savitzky–Golay filter (SG) smoothing. For the quantification of the four phenolic compounds, three different regression models were adopted, namely, SMLR, full-PLS, and Si-PLS, and established, and their results performance was systemically compared and explored. For the quantification of moisture content, the PCR model was constructed.

### 2.6. Evaluation of Model Performance

The performance value of established different models (i.e., SMLR, PLS, Si-PLS, and PCR) was evaluated by four performance indexes including the determination coefficients of calibration (*Rc*^2^), root mean square error of calibration (RMSEC), coefficients of prediction (*Rp*^2^), and root mean square error of prediction (RMSEP). The determination coefficient (*R*^2^) reflects the consistency between the actual and the predictions of the quality parameters, which suggest an performance about the predictive efficiency of the model. The models' efficiency can be concluded by the parameters of RMSEC, RMSEP, and RPD (ratio of standard deviation of the validation set to standard error of prediction). Generally speaking, a high-performance model should yield higher *Rc*^2^ and *Rp*^2^ values but lower RMSEC and RMSEP values.

### 2.7. Software

The samples were divided into two groups consisting calibration and validation sets by the Kennard–Stone algorithm. All data processing of NIR spectra and applications of chemometric methods, including spectral transformation, wavelength/variable selection, and different model construction (SMLR, full-PLS, si-PLS, and PCR), were conducted using TQ Analysis software (version 8.0, Thermo Nicolet, USA). A paired *t*-test was performed to determine if there were differences among the five components' contents obtained by HPLC and in-line NIR analysis using SPSS 17.0 (SPSS Standard version 17.0, SPSS Inc., Chicago, IL), which is a simple and efficient tool for data analysis.

## 3. Results and Discussion

### 3.1. Results of Reference Values Analysis

**Figure 1:**
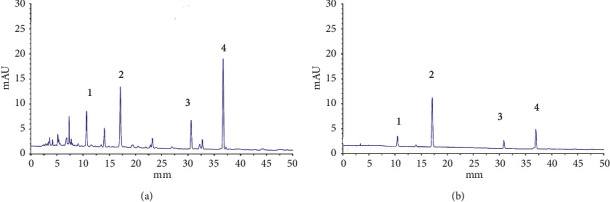
The representative HPLC chromatogram of gastrodin (1), *p-*hydroxybenzyl alcohol (2), parishin B (3), and parishin A (4) in *Gastrodiae rhizoma* steaming samples (A) and standard solution (B).

All samples collected were analyzed using the HPLC method described in Section 2.4. A representative steaming process chromatogram is shown in Figure 1, which reflects that the four phenolic compounds (gastrodin, *p*-hydroxybenzyl alcohol, parishin A, and parishin B) were all baseline separated. The HPLC method was validated before analyzing the samples. The main parameters of the HPLC method are listed in Table 1. The moisture and the concentration of the four analytes in the steaming process samples are listed in Table 2.

The measurement results of four phenolic compounds and moisture in raw *G. rhizoma* showed obvious variation in different samples. The gastrodin, *p*-hydroxybenzyl alcohol, parishin A, and parishin B content ranged from 0.18% to 0.54%, 0.10% to 0.34%, 0.20% to 0.54%, and 0.03% to 0.25%, respectively, with an RSD value of 7.8%, 5.8%, 4.1%, and 3.5%, respectively. Since the raw *G. rhizoma* materials with variable quality due to different geographical sources, harvest times, cultivation conditions and storage, the quality control is essential for steaming process preparation.

### 3.2. Analysis of Near-Infrared Spectra

**Figure 2:**
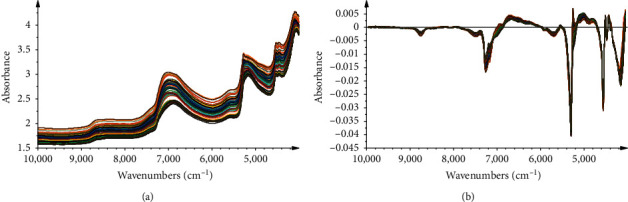
(a) Raw NIR spectra and (b) spectra preprocessed by MSC + FD/SG of all samples collected from the steaming process.

The raw NIR spectra of these collected samples during the steaming process are shown in Figure 2(a), which well monitored the changes in physical and chemical attributes. As the spectra show, the absorbance increased with the process of steaming. While it is generally known that the NIR spectra features with the overtones and combinations of species contain H groups such as -OH, -CH, and -NH, there still exist several characteristic absorption peaks. According to previous studies [23–25], the region from 4200 to 5000 cm^−1^ is implied by the C-H, O-H, and N-H stretch/C-H deformations in the phenyl, since there are several phenyls in the molecular structures of gastrodin, *p*-hydroxybenzyl alcohol, parishin A, and parishin B. In addition, the intense absorptions of the NIR spectra at 5155 cm^−1^ and 6944 cm^−1^ were accounted for the first overtone and deformation of O-H in water [26]. To some extent, the change of spectra in those ranges can describe samples characteristics of the steaming process.

Therefore, the multivariate calibration techniques are useful to reveal the relationship between the NIR spectra and the parameters. Preprocessing of the raw spectra with enhanced signal-to-noise ratios and removal of invalid variations is a necessary step to build high-performance models. To eliminate the baseline drift and scattering effects derived from the inhomogeneous distribution and irregular form of the particles, the first derivative (FD) can be a better selection. To remove the augmentation of noise that derived from the derivatization, the Savitzky–Golay filter algorithm was useful. Finally, in this study, FD with SG smoothing (7th order polynomial, a 5-point window) (FD/SG) was adopted. The spectra processed by FD/SG are shown in Figure 2(b).

### 3.3. Division of Calibration and Validation Sets

All samples were divided into two subsets: the calibration and the prediction subsets. The former was used to establish models and the latter to test the models' accuracy. Initially, spectral outliers were determined by the principal component analysis (PCA) method. According to the original PCA score plots, samples 56, 63, 72, and 141 were abnormal points, which was necessary to eliminate them before model calibration. Then, the Kennard and Stone (K-S) algorithm was adopted to ensure that both sets were well proportioned, which is to cover the multidimensional space in a uniform manner by maximizing the Euclidean distances between already selected objects and the remaining objects. Finally, in about four-fifths of the total samples, 156 were chosen as the calibration set, while the remaining 40 samples were selected as the prediction set. The sample parameters (mean, range, and standard deviation) of the calibration and validation sets are listed in Table 3. It indicates that samples in the calibration and validation sets were distributed appropriately.

### 3.4. Spectral Transformation and Variables Selection

As mentioned above, there are various signal transformation methods that can be used to remove radiation scattering and baseline drift. For example, MSC and SNV are useful for correcting light scattering effects, while FD and SD can eliminate baseline drifts and peak overlap and also can avoid enhancing the noise effect. The performance of four phenolic compounds and moisture calibration with different methods is shown in Table 4, which was evaluated by RMSEC and *Rc*^2^.

The optimization of the spectral transformation methods for NIR models of gastrodin was MSC + SG9 + FD, in which the RMSEC and *Rc*^2^ of the model were 0.0160 and 0.9610; for the NIR models of *p*-hydroxybenzyl alcohol was MSC + SG7 + FD, which the RMSEC and *Rc*^2^ of the model were 0.0165 and 0.9331; for the NIR models of parishin B was MSC + SG7 + FD, in which the RMSEC and *Rc*^2^ of the model were 0.0108 and 0.9245; for the NIR models of parishin A was MSC + SG9 + FD, in which the RMSEC and *Rc*^2^ of the model were 0.0181 and 0.9561; and for the NIR models of moisture was MSC + SG9 + SD, in which the RMSEC and *Rc*^2^ of the model were 1.7 and 0.9513.

### 3.5. Development of Calibration Models

In this study, four different multivariate calibration models including SMLR, full-PLS, si-PLS, and PCR were used to establish the calculated models, and their performance was compared and validated. Specifically, the SMLR model is an early developed regression method that is suitable for the simple system, which performed a better linear relation among different varieties. However, it is prone to be overfitting and lose useful spectrum information. The PCR model was a linear regression model tool that decomposes *X* matric spectrum information, which contains an important step to select best principle factors. Full-PLS is an improved multivariate regression tool that made use of *X* and *Y* matric spectrum information, which was built on the full spectrum. While the Si-PLS model is developed on the different optimal subintervals, which is a subinterval-combination procedure test better than the full-PLS model.

#### 3.5.1. Results of PLS Models

Gastrodin, *p*-hydroxybenzyl alcohol, parishin A, and parishin B were modeled by SMLR, full-PLS, and si-PLS. These models were established using 8 batches as the calibration set and verified by two batches as the validation set. Besides, the latent variables (LVs) were optimized by the leave-one-out method and were determined according to the minimum value of RMSECV. These models' performance was evaluated by the *Rc*^2^ and RMSEC value. The performance of five parameters is shown in Table 5.

As can be seen from Table 5, the SMLR models performed worse as compared with other models, considering that they may lose some important spectrum information to reduce the models' prediction ability. Given this, we selected PLS methods, including full-PLS and Si-PLS models, while the number of principal components (PCs) is critical to the full-PLS models' performance. In this study, the optimum numbers of PCs of the full-PLS models for gastrodin, *p*-hydroxybenzyl alcohol, parishin B, and parishin A were 8, 7, 8, and 6, respectively. The results indicated that the calibration model can be further improved to show a better performance.

**Figure 3:**
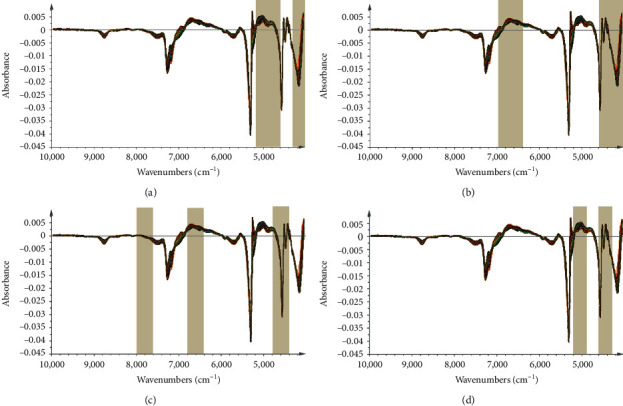
The efficient spectral intervals selected by si-PLS for the prediction of four phenolic compounds: (a) gastrodin, (b) *p*-hydroxybenzyl alcohol, (c) parishin B, and (d) parishin A.

As we know, the Si-PLS models were established based on the combination of different subintervals. Herein, the full spectrum was divided into 10 to 30 intervals. As can be seen from Figure 3, the optimal Si-PLS model of gastrodin by combining 3 subintervals (4,000–4,300 cm^−1^, 4,600–4,900 cm^−1^, and 4,900–5,200 cm^−1^) from 20 intervals was achieved with the highest *Rp*^2^ (0.9591) and lowest RMSEP (0.0181); *p*-hydroxybenzyl alcohol by combining 2 subintervals (4,000–4,600 cm^−1^and 6,400–7,000 cm^−1^) from 10 intervals was achieved with the highest *Rp*^2^ (0.9307) and lowest RMSEP (0.0143); parishin B by combining 3 subintervals (4,400–4,800 cm^−1^, 6,400–6,800 cm^−1^, and 7,600–8,000 cm^−1^) from 15 intervals was achieved with the highest *R*_*p*_^2^ (0.9309) and lowest RMSEP (0.0132); parishin A by combining 2 subintervals (4,300–4,600 cm^−1^ and 4,900–5,200 cm^−1^) from 20 intervals was achieved with the highest *R*_*p*_^2^ (0.9277) and lowest RMSEP (0.0244). In total, the Si-PLS models performed better in the prediction of four phenolic compounds as compared with the SMLR and full-PLS models.

#### 3.5.2. Results of PCR Models

**Figure 4:**
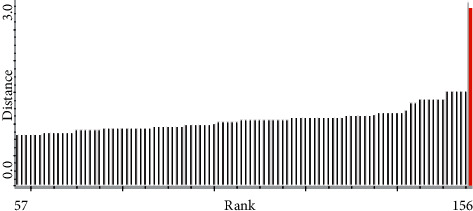
The PCR model of moisture content's spectrum outlier selected according to Mahalanobis distance.

**Figure 5:**
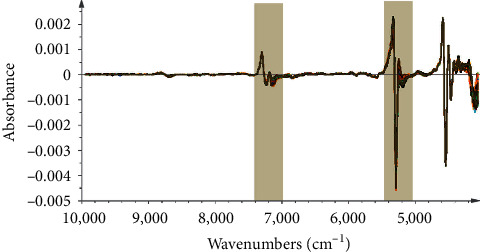
Spectra preprocessed by MSC + SD/SG of all samples and the efficient spectral intervals selected by PCR for the prediction of moisture content.

**Figure 6:**
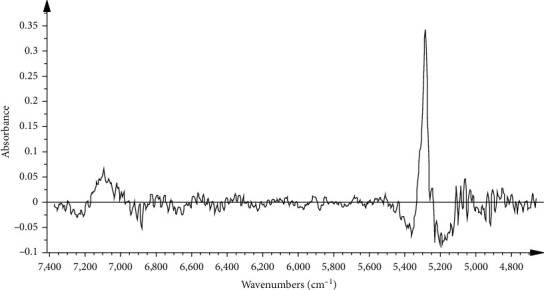
The first loading factor spectra of the PCR model for the moisture content prediction.

**Figure 7:**
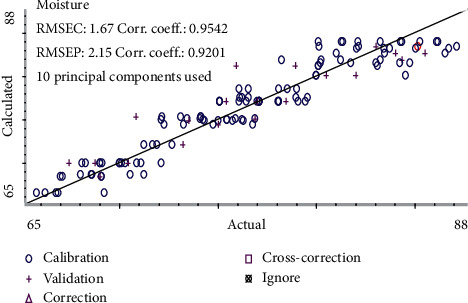
Prediction results of the moisture content models.

The moisture content was modeled by PCR. PCR is a kind of multivariate regression tool, which can map the complex and nonlinear data into a higher dimensional feather space. Some studies have validated that the intense absorptions of the NIR spectra at 5155 cm^−1^ and 6944 cm^−1^ were accounted for the first overtone and deformation of O-H in water [21], and the absorption of former spectra band was stronger than latter. Considering these information for moisture feather spectrum absorption, in this study, these models were first established by 8 batches as the calibration set and verified by two batches as the validation set. The Mahalanobis distance was selected to delete the spectrum outlier in the PCR model in Figure 4. The result suggested that 4 samples should be ignored, and remaining 156 samples were used to establish the calibration model. Then, MSC was chosen as the signal transformation method, taking into account that this signal transformation can reduce the effect of scattered light on diffuse reflection NIR spectra, and SD with SG smoothing (9^th^ order polynomial, a 5-point window) (SD/SG) was adopted in Figure 5. As can be seen from Figure 5, the selected spectral area is related to the moisture content of the sample. It was further confirmed by the fist loading factor of the PCR model from Figure 6, which reflects the main loading information of spectrum. In the end, the performance of the PCR model was evaluated in terms of *Rc*^2^ and RMSEC value. The result of the moisture PCR model is shown in Figure 7.

### 3.6. Validation of Best-Fitted Calibration Models

**Figure 8:**
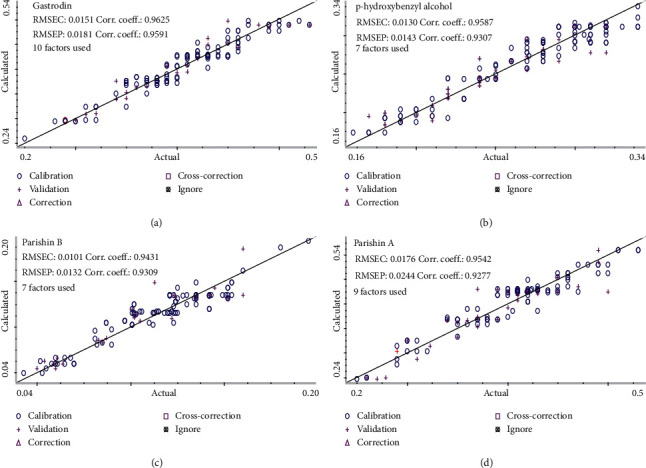
Prediction results of the four PLS models: (a) gastrodin, (b) *p-*hydroxybenzyl alcohol, (c) parishin B, and (d) parishin A.

To predict the content of gastrodin, *p*-hydroxybenzyl alcohol, parishin A, and parishin B and moisture in the steaming process, two batches of 40 samples were selected as the validation set. RMSEP was used as the most critical performance index to evaluate the predictive ability of calibration models. The RMSEP values for above five parameters were sufficiently low, and *R*_*p*_^2^ value was high enough, which means that the models had superior predictive ability. The performance indexes (*R*_*p*_^2^, RMSEP, and RSEP) to evaluate the predictive ability of PLS (full-PLS and Si-PLS) and PCR models are shown in Table 6. Prediction results of the models are shown in Figures 7 and 8. The results show that the established models had a satisfactory predictive ability, and the models could be used to monitor the content of the four analytes and the changes of moisture in the steaming process of *G. rhizoma*.

### 3.7. In-Line and Real-Time Monitoring

In traditional Chinese processing of *G. rhizoma*, steaming is the first step in the manufacturing procedure; however, the variation caused by the batch to batch raw Chinese medicine materials (origin, variety, and so on) and operation environment (equipment, operator, procedure, and so on) could have an effect on the next drying process. In order to improve production efficiency and ensure uniform and controllable quality and in-line and real-time parameter measurements based on PAT technique as NIR would be necessary.

**Figure 9:**
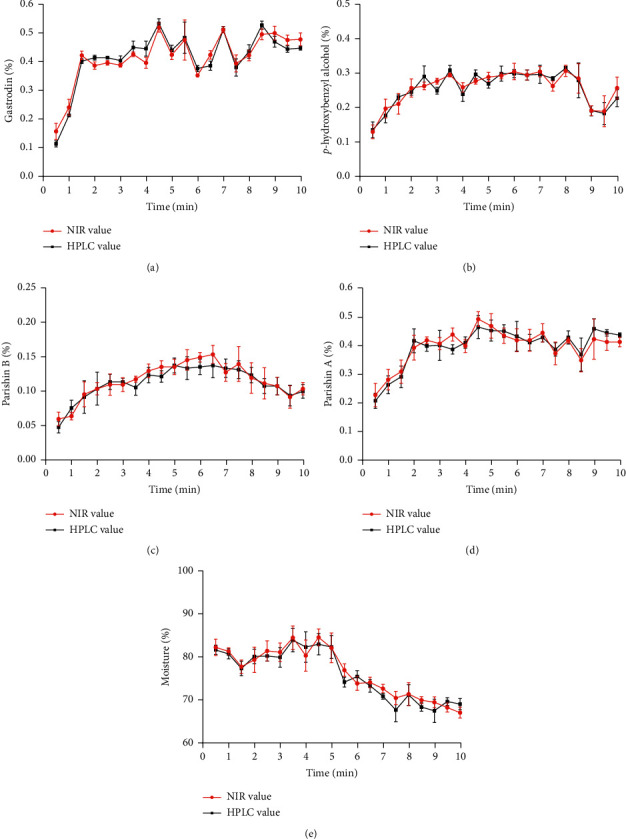
Comparison of the online NIR prediction values with the reference values of the steaming process of the test batch.

In this study, the best-fitted models have been calculated, validated, and uploaded to the above NIR instrument. Then, this NIR method was put into use to monitor the in-line steaming process of three additional batches (provided by the Guizhou Jiulong Group), which were not used before either as a calibration or validation set. Good agreement between gastrodin, *p*-hydroxybenzyl alcohol, parishin A, and parishin B and moisture values predicted by NIR and HPLC was concluded for these additional testing as shown in Figure 9, indicating that these models could be successfully applied in real-time to monitor the steaming process of *G. rhizoma*.

In addition, to evaluate the robustness of the established NIR method, the components' contents obtained by HPLC and in-line NIR analysis were compared using a paired *t*-test in Table 7. Before the paired *t*-test, the variance of both methods was compared using an *F*-test to assess whether they differed significantly, and the results showed that the experimental statistic was lower than the critical (for a significance level of 0.05); thus, it can be concluded that there were no significant differences between the standard deviation of the methods for the sample sets.

Equally, the *t*-experimental statistics were also lower than *t*-critical (for a significance level of 0.05) for five quality control indicators. Thus, the robustness of both methods was comparable, and the established NIR models were considered acceptable for a manufacturing perspective.

Finally, if NIR has been applied to control the endpoint of steaming, the batches would have been released for the next drying step in real-time. While in the traditional Chinese operational mode, the batch operator is waited until time up or depended on the analysis. This “conventional procedure” implied not only a considerable increment in manufacturing, which is time-consuming, laborious, and tedious, but also a potential risk of oversteaming. However, the validated NIR application here is now applied to realize these benefits.

## 4. Conclusions

The steaming process of *G. rhizoma* was stimulated under lab scale in this study, which evaluated the feasibility of the PAT tool NIR spectroscopy approach to improve the quality control efficiency of the process of *G. rhizoma*. First, the reliable and robust NIR quantitative models of gastrodin, *p*-hydroxybenzyl alcohol, parishin A, and parishin B and moisture in the steaming process were established and validated, and the proposed algorithm Si-PLS was superior to other models. Then, the established best-fitted models were in real-time applied to the release test of *G. rhizoma*, which can guarantee stable and reliable quality of the steaming process. Additionally, compared to the traditional method such as HPLC, this nondestructive and rapid technique could offer significant advantages, especially in aspect of improving the stability and uniformity of the product, which is beneficial to industrial factory. Overall, the results of this study showed that NIR technique coupled with the multivariate regression tool could be applied successfully for real-time and in-line measurements of the steaming process of *G. rhizoma* on an actual industrial scale. In addition, since these results preliminarily demonstrated that NIR technique coupled with the multivariate regression tool could be applied successfully for real-time and in-line measurements of the steaming process of *G. rhizoma*, more samples will be needed to confirm these results and develop more robust models for prediction in the further study.

## Figures and Tables

**Table 1:** Main methodology parameters and calibration curves of the reference HPLC method.

Analytes	Linearity ranges (mg/mL)	Calibration curves	*R* ^2^	Repeatability (RSD %, *n* = 6)	Recovery (%, *n* = 6)
Gastrodin	0.0024–0.1478	*Y* = 780.37*X* – 4.1049	0.9999	0.23	99.8
*p-*Hydroxybenzyl alcohol	0.0055–0.0825	*Y* = 3112.3*X* – 5.5707	0.9999	0.15	100.5
Parishin B	0.0015–0.0300	*Y* = 7095*X* – 9.9625	0.9997	0.09	101.2
Parishin A	0.0016–0.1260	*Y* = 1478*X* + 6.6621	0.9998	0.12	98.6

**Table 2:** Results of four analytes and moisture contents of 200 samples.

Batch	Range	Gastrodin (mg/g)	*p-*Hydroxybenzyl alcohol (mg/g)	Parishin B (mg/g)	Parishin A (mg/g)	Moisture (%)
1	Max	6.2648	3.4677	1.5923	5.8750	89.12
	Min	0.1898	0.4819	0.1299	0.1241	65.43
2	Max	6.0611	3.6677	1.4911	5.9755	85.72
	Min	0.1921	0.4112	0.1093	0.1148	66.23
3	Max	6.4611	3.6642	1.5943	5.8720	89.02
6	Max	6.2601	3.0672	1.6001	5.9733	81.02
	Min	0.1468	0.4116	0.1093	0.1135	68.03
7	Max	6.6108	3.1637	1.6023	5.8030	83.02
	Min	0.1291	0.4122	0.1098	0.1142	66.43
8	Max	6.6611	3.5112	1.5311	5.9112	86.02
	Min	0.1456	0.4013	0.1091	0.1432	68.23
9	Max	6.2018	3.4577	1.6113	5.6510	82.42
	Min	0.1458	0.4249	0.1019	0.1098	68.03
10	Max	6.4562	3.4112	1.5879	5.8670	81.12
	Min	0.1098	0.4342	0.1256	0.1230	66.43

**Table 3:** Reference results of samples in calibration and validation sets.

Parameters	Units	Subsets	S.N	Range	Mean	S.D
Gastrodin	%	Calibration set	156	0.20–0.53	0.32	8.1
		Validation set	40	0.18–0.54	0.35	7.9
*p-*Hydroxybenzyl alcohol	%	Calibration set	156	0.10–0.34	0.25	2.5
		Validation set	40	0.12–0.32	0.23	2.9
Parishin B	%	Calibration set	156	0.03–0.25	0.12	1.9
		Validation set	40	0.05–0.21	0.14	1.8
Parishin A	%	Calibration set	156	0.20–0.54	0.39	2.0
		Validation set	40	0.21–0.53	0.38	2.5
Moisture	%	Calibration set	156	65.03–89.12	78.15	5.6
		Validation set	40	66.43–88.15	76.43	6.2

S.N, the number of samples; S.D, standard deviation.

**Table 4:** Results of different spectral transformation for gastrodin, *p-*hydroxybenzyl alcohol, parishin A, and parishin B models.

Parameters	Pretreatment	RMSEC	*Rc* ^2^	LVs
Gastrodin	Raw	0.0168	0.9561	8
	MSC	0.0158	0.9598	7
	MSC + SG9	0.0157	0.9601	9
	MSC + SG9 + FD	0.0160	0.9610	10
	MSC + SG9 + SD	0.0185	0.9469	7

*p-*Hydroxybenzyl alcohol	Raw	0.0198	0.9120	7
	MSC	0.0161	0.9234	6
	MSC + SG7	0.0151	0.9301	6
	MSC + SG7 + FD	0.0165	0.9331	5
	MSC + SG7 + SD	0.0168	0.9312	5

Parishin B	Raw	0.0198	0.9110	7
	MSC	0.0165	0.9126	6
	MSC + SG7	0.0143	0.9178	6
	MSC + SG7 + FD	0.0108	0.9245	6
	MSC + SG7 + SD	0.0113	0.9211	5

Parishin A	Raw	0.0198	0.9433	8
	MSC	0.0189	0.9511	9
	MSC + SG9	0.0186	0.9532	9
	MSC + SG9 + FD	0.0181	0.9561	9
	MSC + SG9 + SD	0.0191	0.9598	8
	Raw	2.72	0.8713	5
	MSC	2.99	0.8421	5

Moisture	MSC + SG9	2.72	0.8467	5
	MSC + SG9 + FD	1.87	0.9132	6
	MSC + SG9 + SD	1.71	0.9513	6

**Table 5:** Results of different models for predicting gastrodin, *p-*hydroxybenzyl alcohol, parishin B, and parishin A.

Parameters	Methods	PCs	Calibration		Validation	
			*Rc* ^2^	RMSEC	*Rp* ^2^	RMSEP
Gastrodin	SMLR	2	0.5163	0.0475	0.6505	0.0513
	si-PLS	9	0.9625	0.0151	0.9591	0.0181
	Full-PLS	8	0.9112	0.1019	0.9088	0.1026

*p-*Hydroxybenzyl alcohol	SMLR	6	0.7959	0.0277	0.8472	0.0210
	si-PLS	9	0.9587	0.0130	0.9307	0.0143
	Full-PLS	7	0.8775	0.0148	0.8622	0.0154

Parishin B	SMLR	5	0.8903	0.0128	0.8219	0.0112
	si-PLS	9	0.9431	0.0101	0.9309	0.0132
	Full-PLS	8	0.9154	0.0198	0.9025	0.0215

Parishin A	SMLR	5	0.5720	0.0482	0.5234	0.0558
	si-PLS	9	0.9542	0.0176	0.9277	0.0244
	Full-PLS	6	0.9235	0.0365	0.9354	0.0422

**Table 6:** Statistics of the optimal calibration models for five indicators.

Quality control indicators	Calibration set			Validation set			LVs
	*Rc* ^2^	RMSEC	RMSECV	*Rp* ^2^	RMSEP	RPD	
Gastrodin	0.9625	0.0151	0.0232	0.9591	0.0181	4.85	10
*p-*Hydroxybenzyl alcohol	0.9587	0.0130	0.0125	0.9307	0.0143	3.80	7
Parishin B	0.9431	0.0101	0.0112	0.9309	0.0132	3.80	7
Parishin A	0.9542	0.0176	0.0182	0.9277	0.0244	3.72	9
Moisture	0.9542	1.67	1.8760	0.9201	2.15	3.54	10

**Table 7:** Statistics of five indicators' comparison between HPLC and NIR methods using a paired *t*-test.

Quality control indicators	Method	Number of samples	Mean	Variance	*P* value	
					*F*-test	*t*-test
Gastrodin	HPLC	60	0.41	0.089	0.899	0.905
	NIR	60	0.42	0.099		
*p-*Hydroxybenzyl alcohol	HPLC	60	0.26	0.052	0.162	0.170
	NIR	60	0.25	0.055		
Parishin B	HPLC	60	0.12	0.0298	0.078	0.085
	NIR	60	0.11	0.0259		
Parishin A	HPLC	60	0.40	0.065	0.925	0.974
	NIR	60	0.39	0.069		
Moisture	HPLC	60	76.56	5.987	0.425	0.469
	NIR	60	76.33	5.995		

## Data Availability

The data used to support the results of this study are included within the article. Any further information is available from the authors upon request.

## References

[1] Zhan H. D., Zhou H. Y., Sui Y. P. (2016). The rhizome of Gastrodia elata Blume—an ethnopharmacological review. *Journal of Ethnopharmacology*.

[2] Tang C., Wu B., Wu J. (2018). Novel strategies using total gastrodin and gastrodigen in, or total gastrodigen in for quality control of Gastrodia elata. *Molecules*.

[3] Baek N. I., Choi S. Y., Park J. K. (1999). Isolation and identification of succinic semialdehyde dehydrogenase inhibitory compound from the rhizome of Gastrodia elata blume. *Archives of Pharmacal Research*.

[4] Jeon J.-S., Kim J., Park S., Ryou C., Kim C. Y. (2016). Preparative purification of plasmin activity stimulating phenolic derivatives fromGastrodia elata using centrifugal partition chromatography. *Biomedical Chromatography*.

[5] Dai R., Wang T., Si X. (2017). Vasodilatory effects and underlying mechanisms of the ethyl acetate extracts from Gastrodia elata. *Canadian Journal of Physiology and Pharmacology*.

[6] Chinese Pharmacopoeia Commission (2015). *Pharmacopoeia of the People’s Republic of China*.

[7] The Society of Japanese Pharmacopoeia (2015). *Japanese Pharmacopoeia Committee, the Japanese Pharmacopoeia*.

[8] Qian Y. F., Shang E. X., Duan J. A. (2012). Construction of a rapid identification method for chemical constituents in traditional Chinese medicine based on liquid chromatography-hybrid mass spectrometry-database. *Zhongguo Zhong Yao Za Zhi*.

[9] Liu Y., Huang G. (2018). The content analysis of gastrodin and gastrodigenin obtained by different processing methods. *Journal of Chromatographic Science*.

[10] Li Z., Wang Y., Ouyang H. (2015). A novel dereplication strategy for the identification of two new trace compounds in the extract of Gastrodia elata using UHPLC/Q-TOF-MS/MS. *Journal of Chromatography B*.

[11] Lee D. K., Lim D., Um J. (2014). Evaluation of four different analytical tools to determine the regional origin of Gastrodia elata and Rehmannia glutinosa on the basis of metabolomics study. *Molecules*.

[12] Al-Taweel A. M., Abdel-Kader M. S., Fawzy G. A. (2015). Isolation of flavonoids from Delonix elata and determination of its rutin content using capillary electrophoresis. *Pakistan Journal of Pharmaceutical Sciences*.

[13] Saerens L., Dierickx L., Quinten T. (2012). In-line NIR spectroscopy for the understanding of polymer-drug interaction during pharmaceutical hot-melt extrusion. *European Journal of Pharmaceutics and Biopharmaceutics*.

[14] Li Y., Liu B., Geng S. (2016). An approach combining real-time release testing with near-infrared spectroscopy to improve quality control efficiency of Rhizoma paridis. *Spectrochimica Acta Part A: Molecular and Biomolecular Spectroscopy*.

[15] Yang Y., Wang L., Wu Y. (2017). On-line monitoring of extraction process of Flos Lonicerae Japonicae using near infrared spectroscopy combined with synergy interval PLS and genetic algorithm. *Spectrochimica Acta Part A: Molecular and Biomolecular Spectroscopy*.

[16] Peinado A., Hammond J., Scott A. (2011). Development, validation and transfer of a near infrared method to determine in-line the end point of a fluidised drying process for commercial production batches of an approved oral solid dose pharmaceutical product. *Journal of Pharmaceutical and Biomedical Analysis*.

[17] Ma X. D., Fan Y. X., Jin C. C. (2016). Specific targeted quantification combined with non-targeted metabolite profiling for quality evaluation of Gastrodia elata tubers from different geographical origins and cultivars. *Journal of Chromatography A*.

[18] Zhu R. G., Yao X. D., Duan H. W. (2016). Study on the rapid evaluation of total volatile basic nitrogen (TVB-N) of mutton by hyperspectral imaging technique. *Guang Pu Xue Yu Guang Pu Fen Xi*.

[19] Chen H., Tan C., Lin Z., Wu T. (2018). Classification and quantitation of milk powder by near-infrared spectroscopy and mutual information-based variable selection and partial least squares. *Spectrochimica Acta Part A: Molecular and Biomolecular Spectroscopy*.

[20] Ghasemi-Varnamkhasti M., Mohtasebi S. S., Rodriguez-Mendez M. L., Gomes A. A., Araújo M. C. U., Galvão R. K. H. (2012). Screening analysis of beer ageing using near infrared spectroscopy and the Successive Projections Algorithm for variable selection. *Talanta*.

[21] Lu W. Z. (2007). *Modern Near Infrared Spectroscopy Analytical Technology*.

[22] Zuo Y., Deng X., Wu Q. (2018). Discrimination of gastrodia elata from different geographical origin for quality evaluation using newly-build near infrared spectrum coupled with multivariate analysis. *Molecules*.

[23] Burns D. A., Ciurczk E. W. (2007). *Handbook of Near-Infrared Analysis*.

[24] Workman J., Weyer L. (2008). *Practical Guide to Interpretive Near-Infrared Spectroscopy*.

[25] Liu K.-Z., Shi M., Man A., Dembinski T. C., Shaw R. A. (2005). Quantitative determination of serum LDL cholesterol by near-infrared spectroscopy. *Vibrational Spectroscopy*.

[26] Blanco M., Peguero A. (2008). An expeditious method for determining particle size distribution by near infrared spectroscopy: comparison of PLS2 and ANN models. *Talanta*.

